# Low-volume Nordic hamstring training improves change-of-direction performance more than higher volumes in youth soccer players

**DOI:** 10.3389/fspor.2026.1818507

**Published:** 2026-07-03

**Authors:** Raouf Hammami, Javier Gene-Morales, Pablo Jiménez-Martínez, Abdelkader Mahmoudi, Haithem Rebai, Carlos Alix-Fages, Amador García-Ramos, Danica Janicijevic, Juan C. Colado

**Affiliations:** 1 Tunisian Research Laboratory ‘Sports Performance Optimization’, National Center of Medicine and Science in Sports (CNMSS) (CNMSS-LR09SEP01), Tunis, Tunisia; 2 Higher Institute of Sport and Physical Education of Ksar-Said, Manouba University, Manouba, Tunisia; 3 Research Group in Prevention and Health in Exercise and Sport (PHES), Department of Physical Education and Sports, University of Valencia, Valencia, Spain; 4 Department of Health Research, ICEN Cognis, Santa Cruz de Tenerife, Spain; 5 Department of Physical Education and Sport, Faculty of Sport Sciences, University of Granada, Granada, Spain; 6 Department of Sports Sciences and Physical Conditioning, Faculty of Education, Universidad Católica de la Santísima Concepción, Concepción, Chile; 7 Research Academy of Human Biomechanics, The Affiliated Hospital of Medical School of Ningbo, Ningbo, China; 8 Faculty of Sports Science, Ningbo University, Ningbo, China

**Keywords:** agility, dose-response, eccentric strength, football, speed

## Abstract

**Introduction:**

Soccer players adopt hamstring-strengthening programs to enhance sprint and change-of-direction (CoD) performance, despite limited information on optimal volume. Therefore, we aimed to compare low, moderate, and high volumes of Nordic curl training on linear sprint and CoD in pubertal soccer players.

**Methods:**

Sixty male youth soccer players (13.69 ± 0.31years, 159.43 ± 8.55 cm, 48.51 ± 7.93 kg) were randomly assigned into three groups: low- (*n* = 20; 4 sets of 3 repetitions), moderate- (*n* = 20; 4 sets of 6 repetitions), and high-volume Nordic curl training (*n* = 20; 4 sets of 9 repetitions). The training program lasted 8 weeks in-season. Assessments of sprinting (10 and 20 meters) and CoD performance (15-m-dribbling test with and without a ball) were conducted before and after the intervention.

**Results:**

While all three groups improved their performance across all tests, a two-way repeated-measures ANCOVA revealed a significant time × group interaction (*p* = 0.012) for the 10 m linear sprint, with low- (*p* = 0.022, *d* = 0.78) and moderate-volume (*p* = 0.055, *d* = 0.71) Nordic curl training yielding better results than high-volume Nordic curl training. Nonsignificant between-group differences were encountered for the 20 m sprints. A significant time × group interaction was observed for the CoD without (*p* = 0.002) and with the ball (CoDB, *p* = 0.014), with significantly better results for the low-volume Nordic curl training compared to moderate- (CoD, *p* = 0.012, *d* = 0.59; CoDB, *p* = 0.021, *d* = 0.60) and high-volume (CoD *p* = 0.004, *d* = 0.78; CoDB, *p* = 0.042, *d* = 0.56) Nordic curl training.

**Discussion:**

Low-volume Nordic curl training is the most effective training dose for improving CoD performance in top-tier youth soccer athletes. For sprint, both low and moderate volumes improve the 10 m sprint more than high volume, and all three volumes improve the 20 m sprint similarly.

## Introduction

Soccer entails high-intensity movements such as changing directions with or without the ball, sprinting, jumping, or kicking, combined with relatively low-intensity actions (1–4). This unpredictable combination, along with the significant demands and loads that soccer players must meet during the season, makes soccer a team sport with a high risk of injury (5). For instance, during the late swing phase of running, the hamstrings contract eccentrically to decelerate the forward movement of the foot and leg, as in sprints and change-of-direction (CoD) tasks present in soccer (6, 7). In addition, during CoD tasks, the deceleration phase is shortened, increasing the risk of tears in the muscle-tendon unit and requiring greater hamstrings eccentric activation to compensate for forward momentum (8). For this study, we will refer to CoD rather than agility, as the latter includes reacting to an external stimulus (9). Although Nordic eccentric hamstring training has been mainly studied for injury prevention, it also increases speed, acceleration, CoD muscle fascicle length, strength (7, 10–15), and may improve torque transmission across the lower-limb kinetic chain (10, 16).

Nordic curl training programs usually entail long durations (e.g., 13 weeks) and high training volumes (2–3 sessions/week, up to 30 repetitions/session), which hinder their implementation among elite youth athletes, especially during season (17). For instance, the study by Mjølsnes et al. (6) first introduced the Nordic curl eccentric hamstring exercise, with a volume of 700 repetitions distributed over 10 weeks, resulting in greater hamstring strength than the concentric hamstring curl. However, the Nordic curl involves an eccentric action during which myosin cross-bridges are already bound to actin and separate as the sarcomeres lengthen, thereby increasing muscle damage (18); therefore, a lower training volume may allow for better recovery. Furthermore, due to the maximal eccentric effort and high dosages prescribed, compliance with Nordic Hamstring training protocols in top-tier soccer teams is small despite a level of incidence of hamstring strain injuries (19). In this regard, high volumes and players' recurring complaints of delayed-onset muscle soreness are among the reasons for low compliance with Nordic curl interventions among 50 clubs from the Union of European Football Associations (UEFA) Champions League (20).

Shorter programs with less training volume can be easier to implement within the demanding, competitive calendars of top-tier teams (19), although it could limit strength gains (21). Previous research has shown that using high and low volumes for Nordic training are similarly effective in increasing strength, jump, and speed in female football athletes (10). To date, evidence supporting lower doses of Nordic curl on athletes' performance is scarce (19). To the best of our knowledge, three sets of three repetitions thrice per week, totaling 27 repetitions/week, is the minimum volume studied for a Nordic curl training protocol (19), with no previous research evaluating 2-day-per-week programs with fewer than 27 weekly repetitions in youth elite athletes. Therefore, although a minimum volume threshold has not yet been identified, it is important to compare how different volume ranges affect sprint and CoD performance in youth soccer players.

This study aimed to identify adaptations in linear sprint performance (10 and 20 m) and in CoD, both without the ball (CoD) and with the ball (CoDB), following an 8-week Nordic curl training program of three different volumes (low, moderate, and high) in youth soccer players. According to prior literature (10, 16, 19), we expected that low-volume training would produce superior adaptations compared to medium- and high-volume training.

## Materials and methods

The study participants were recruited from the professional soccer club: “Stade Tunisien, Bardo, Tunisia”. Prior to starting, participants received a document detailing the scope, aims, procedures, benefits, and risks of the research. Legal guardians and the participants provided informed consent after carefully reading this document and clarifications. We followed the latest version of the Declaration of Helsinki. Before the experimental testing, we obtained approval from the National Centre of Medicine and Science of Sports of Tunis Local Ethics Committee (CNMSS-LR09SEP01).

### Participants

Sixty male youth soccer players aged 13–14 years participated. Participants' features are shown in Table 1. The players came from families of similar socio-economic backgrounds, followed identical daily school schedules, and were players of the same club. None of them presented a record of musculoskeletal, neurological, or orthopedic conditions affecting their participation in the study. A control group was not included because all the experimental groups comprised top-tier players competing at national level, and no comparable athletes with similar preintervention levels for sprint and CoD could be recruited, as happened in previous research (22, 23).

**Table 1:** Anthropometric characteristics of each group of Nordic hamstring training.

Variables/Groups	High volume (*n* = 20)	Moderate volume (*n* = 20)	Low volume (*n* = 20)	Between-group differences
Age (years)	13.67 ± 0.32	13.67 ± 0.32	13.70 ± 0.30	F = 0.13, *p* = 0.879
Height (cm)	157.10 ± 7.97	159.75 ± 9.28	161.45 ± 8.20	F = 1.33, *p* = 0.273
Body mass (kg)	46.90 ± 6.68	47.72 ± 8.591	50.90 ± 8.21	F = 1.44, *p* = 0.245
MO^a^ (years)	−0.09 ± 0.43	−0.09 ± 0.43	0.00 ± 0.35	F = 0.28, *p* = 0.756
APHV^b^ (years)	13.63 ± 0.54	13.63 ± 0.54	13.71 ± 0.45	F = 0.18, *p* = 0.837

Data are presented as mean ± standard deviation.

a MO, maturity offset.

b Age at peak height velocity.

### Procedures

**Figure 1:**
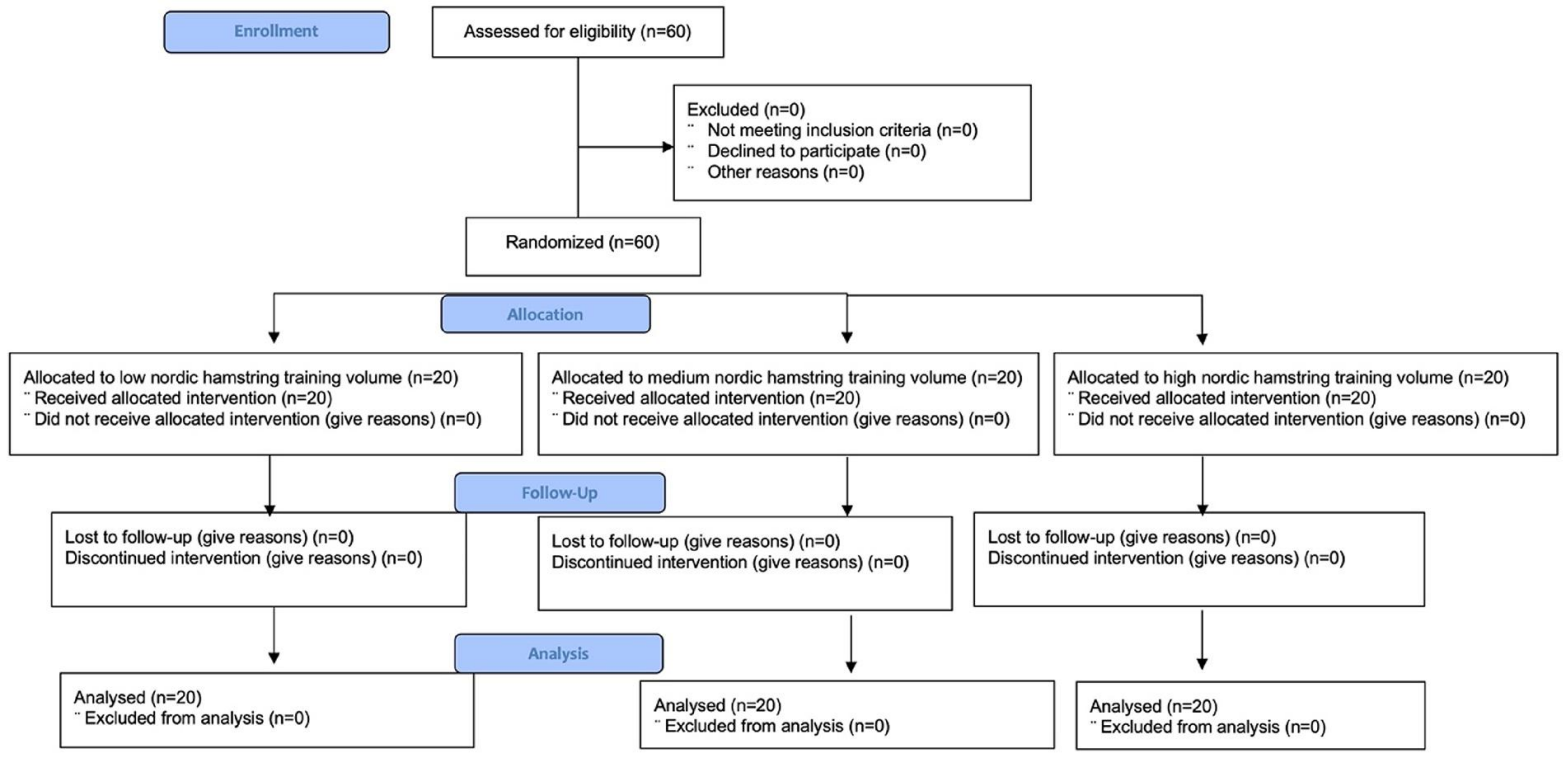
Flow diagram (the CONSORT: consolidated standards of reporting trials).

All the procedures were performed during the second half of the competitive soccer season (February and March 2022). Figure 1 presents the participant flow throughout the study. Participants were randomly assigned to three Nordic curl training subgroups: Low-volume Nordic curl training (*n* = 20), Medium-volume Nordic curl training (*n* = 20), and High-volume Nordic curl training (*n* = 20). Prior to starting the experimental procedures, all players completed two familiarization sessions during which the procedures were explained and carried out. For this purpose, participants were shown examples of how to perform each test and the Nordic curl. Afterward, they performed each test at a submaximal speed once, followed by two sets of one repetition of the Nordic curl. Pre-and post-intervention sprint and CoD testing was conducted. Testing began five minutes after a 15 min general warm-up consisting of submaximal-intensity running, calisthenic exercises (bodyweight squats, multiplanar lunges, inchworms, Spiderman planks, and Spiderman walks), and dynamic lower-limb stretching.

### Instruments and protocols

#### Anthropometrics

The height and body mass of all participants were measured during the pre-testing session using a stadiometer (Holtain Limited, Crosswell, Wales, UK) and an electronic scale (Scale Electronics Development, NY, USA). We estimated the maturity offset following previous research by Moore et al. (24). We measured leg length with a tape from the anterior superior iliac spine (ASIS) to the most distal part of the medial malleolus with the participant lying supine (25, 26).

#### Linear sprint

We evaluated running speed in seconds over a 20-meter sprint, from which times for 10 and 20 meters were extracted. The athletes were instructed to sprint at maximum speed from a static standing position. The participants were 20 cm behind the first timing gate. with the dominant foot backward and minimal torso lean. We used Brower Timing Systems photocells (Salt Lake City, UT, USA) with an accuracy of 0.01 s, placed 0.4 meters above the ground, to automatically record the time. The athletes were allowed two attempts, with a two-minute passive rest between attempts. The best performance was kept for further investigation.

#### Agility (15 m-dribbling CoD test)

The athletes started the run three meters behind the first photocells, they run straight for 3 meters, performed a slalom section of three meters marked by three sticks of 1.6 meters positioned in line and separated by 1.5 meters each, then they jump a hurdle of 0.5 meters height placed 2.0 m beyond the third stick. Finally, players ran 7 m to overcome the second set of photocell gates (Brower Timing Systems, Salt Lake City, UT, USA), which stopped the timer (27). The test was performed first without a ball and then with ball (28). This CoD test presents excellent test-retest reliability [intraclass correlation coefficients (ICCs): 0.93–0.97] (29), measured both with and without a ball. Participants performed two attempts, with a three-minute passive rest between them. The best performance was kept for further analysis.

#### Training program

All participants were randomly assigned to high-, medium-, or low-volume Nordic curl training in addition to their regular soccer preparation (Table 2). We designed the training intervention following previously published research on Nordic curl training 19(15). The Nordic curl training program lasted 8 weeks and was performed before team training, which was scheduled on Tuesdays and Thursdays. Apart from the Nordic hamstring exercise protocol, no additional lower-limb strength training was prescribed during the intervention period. Participants maintained their regular sport-specific training routines.

**Table 2:** Design of the high, medium, and low Nordic hamstring training programs in youth soccer players.

	High volume	Moderate volume	Low volume
Total repetitions	576 study total	384 study total	192 study total
	72 per week	48 per week	24 per week
	36 per day	24 per day	12 per day
Day 1 (sets × repetitions)	4 × 9	4 × 6	4 × 3
Day 2 (sets × repetitions)	4 × 9	4 × 6	4 × 3

To perform Nordic curl training, the athletes started kneeling, feet perpendicular to the lower leg (90° ankle joint angle), with another participant holding their ankles in position by leaning on to provide stability and avoid compensation. Participants then eccentrically extended the knees with the lower leg as the fixed segment, i.e., lowered the body while keeping the head, spine, and hips neutral, arms crossed over the chest, palms on the shoulders. The aim was to slow the movement through the action of the posterior chain, mainly the hamstrings and gluteus, while using the arms at the end of the range of motion to absorb the impact. From there, they pushed hard with their arms to return to the initial position, then repeated the movement for the prescribed number of repetitions. While resting, participants held another team member's legs, swapping roles to ease the procedures. Therefore, at least one minute of rest was allowed between sets (30). We controlled the technique as much as possible. When players were fatigued, a minimum rest period was allowed between reps to allow them to recover and maintain proper technique.

#### Statistical analyses

We carried out all statistical analyses in SPSS (v. 28; IBM Corp., Armonk, NY, USA), with *p* < 0.05 as the threshold for statistical significance. After basic data curation, we used the Kolmogorov and Levene tests to assess the normality and homoscedasticity, respectively. We transformed non-normal variables into normal with the so-called Two-Step Procedure (31). All variables met the homoscedasticity assumption. After preliminary normality assessment and data curation, an exploratory correlational analysis and comparisons between pre-PHV and post-PHV athletes were conducted to evaluate the potential influence of maturity offset on training adaptations. Given that we obtained non-significant results, the maturity offset was treated only as a sample feature. At this point, we examined and found between-group differences in baseline levels of the dependent variables using a one-way analysis of variance (ANOVA). Therefore, we chose a two-way analysis of covariance (ANCOVA) for repeated measures; time (pre-intervention and post-intervention) and training volume (high, medium, and low) were included as the within- and between-subjects factors, respectively. The baseline measurement of each variable was used as a covariate. We reported the effect size as partial eta squared (ƞp²), with 0.01 < ƞp² < 0.06 indicating a small effect, 0.06 ≤ ƞp² ≤ 0.14 indicating a medium effect, and ƞp² > 0.14 indicating a large effect. Afterward, *post-hoc* tests were conducted with the Bonferroni adjustment to identify statistically significant differences. For this comparisons, we reported the effect size as the Cohen's d (32), which was interpreted according to specific guidelines for Sport Sciences (33), in which *d* < 0.20 represents a trivial effect size, *d* = 0.20–0.59 represents a small effect size, *d* = 0.60–1.19 represents a moderate effect size, *d* = 1.20–1.99 represents a large effect size, *d* = 2.00–3.99 represents a very large effect size, and *d* ≥ 4.00 represents a nearly perfect effect size.

We estimated the required sample size with the software G*Power (v. 3.1.9.6), configured as follows: *F*-tests–Repeated measures mixed, 2 measurement points, 3 groups. We used an alpha level of 0.05, 90% power, a correlation among repeated measures of 0.5, and an effect size of *f* = 0.25, which is considered small. The software showed that at least 54 athletes were needed.

## Results

All initially involved players completed all the study procedures, including evaluations, familiarization, and training. No adverse events were reported. All the study groups had statistically similar ages, maturity, and anthropometric measures (Table 1).

### Linear sprint time

Two-way repeated-measures ANCOVA revealed that time [F(1,56) = 4.98, *p* = 0.030, ƞp² = 0.08] and time × group interaction [F(2, 56) = 4.79, *p* = 0.012, ƞp² = 0.15] significantly influenced the 10 m sprint. The time [F(1,56) = 4.11, *p* = 0.047, ƞp² = 0.07] but not the time × group interaction [F(2,56) = 2.81, *p* = 0.068, ƞp² = 0.09], showed a significant effect on the 20 m sprint.

Bonferroni *post-hoc* tests exhibited significant between-group differences with moderate effect sizes in the 10 m sprint, with worse results for the High compared to Low (*p* = 0.022, *d* = 0.78) and High to Medium (*p* = 0.055, *d* = 0.71); the comparison between the Low and Moderate was not significant (*p* = 0.999). All the groups showed statistically similar outcomes in the 20 m sprint (*p* = 0.084–0.999). Table 3 presents the sprint results, adjusted for the covariate, for each group.

**Table 3:** Effects of different Nordic hamstring training volumes (high, moderate, and low) on linear sprint time in youth soccer players.

Variable	Time	1. High volume		2. Moderate volume		3. Low volume	
		Mean	SD	Mean	SD	Mean	SD
10-meter sprint (seconds)	Pre	2.028		2.028**		2.028***	
	Post	2.037^†^	0.076	1.982	0.071	1.956	0.089
20-meter sprint (seconds)	Pre	3.419*		3.419**		3.419***	
	Post	3.355	0.121	3.341	0.112	3.252	0.138

Data are presented as mean and standard deviation (SD).

* Significant difference (*p* < 0.05) between pre-and post-intervention measurements.

** Highly significant difference (*p* < 0.01) between pre-and post-intervention measurements.

*** Very highly significant difference (*p* < 0.001) between pre-and post-intervention measurements.

† Significant differences (*p* < 0.05) with the Group 2 (moderate-volume Nordic hamstring training), or 3 (low-volume Nordic hamstring training), respectively.

### Cod outcomes

Two-way repeated-measures ANCOVA showed that the time and time × group interaction significantly influenced the CoD [F(1,56) = 8.45, *p* = 0.005, ƞp² = 0.13; F(2, 56) = 6.84, *p* = 0.002, ƞp² = 0.20] and CoDB [F(1,56) = 7.75, *p* = 0.007, ƞp² = 0.12; F(2, 56) = 4.62, *p* = 0.014, ƞp² = 0.14].

Bonferroni *post-hoc* test showed that the Low improved the CoD and CoDB significantly more than High and Moderate (CoD, Low-Moderate: *p* = 0.012, *d* = 0.59, Low-High: *p* = 0.004, *d* = 0.78; CoDB, Low-Moderate: *p* = 0.021, *d* = 0.60, Low-High: *p* = 0.042, *d* = 0.56). The High and Moderate showed statistically similar outcomes (*p* = 0.999). Table 4 presents the CoD results, adjusted for the covariate, for each group.

**Table 4:** Effects of different Nordic hamstring training volumes (high, moderate, and low) on change-of-direction outcomes in youth soccer players.

Variable	Time	1. High volume		2. Moderate volume		3. Low volume	
		Mean	SD	Mean	SD	Mean	SD
CoD (seconds)	Pre	4.213*		4.213**		4.213**	
	Post	4.092***	0.259	4.025***	0.233	3.802	0.246
CoDB (seconds)	Pre	5.201**		5.201**		5.201**	
	Post	4.949***	0.282	4.951***	0.264	4.707	0.286

Data are presented as mean and standard deviation (SD). CoD, change of direction without ball; CoDB, change of direction with ball.

* Significant difference (*p* < 0.05) between pre-and post-intervention measurements.

** Very highly significant difference (*p* < 0.001) between pre-and post-intervention measurements.

*** Significant differences (*p* < 0.05) with the low volume of Nordic hamstring training.

## Discussion

This study aimed to identify adaptations in linear sprint performance (10 and 20 m) and CoD following an 8-week Nordic curl training program at three volumes (low, moderate, and high) in youth soccer athletes. In agreement with the study hypothesis, the most noteworthy finding was that a low-volume Nordic curl training prescription over 8 weeks can produce moderate improvements in CoD performance, with and without the ball, compared with a higher-volume prescription. Additionally, the low- and moderate-volume programs showed significantly better results than the high-volume program in the 10 m sprint, and no between-group differences were observed for the 20 m sprint. Herein, we compare and assess these findings in light of prior empirical evidence.

First, previous research, including a meta-analysis, confirms similar findings across different volumes of Nordic curl training programs, concluding that lower volumes do not attenuate adaptations (10, 16, 19). Similarly, Lacome et al. (16) suggest that low-volume knee-flexor eccentric training helps maintain muscle adaptations, regardless of prior high-volume training. Besides, regular use of microdoses of eccentric exercises at high intensities appears to be beneficial for preventing injuries (34). By contrast, Razzaq et al. (35) showed that higher-volume Nordic curl training elicited greater improvements than low- or moderate-volume training modalities. Based on our findings and previous research, the total volume per session does not appear to be critical for achieving adaptations in muscle parameters such as fascicle length or knee-flexor strength (14, 15), and in performance measured by sprint and CoD.

Greater enhancement of the 10 m linear sprint was observed following the low- and moderate-volume Nordic curl training in comparison to the high-volume Nordic curl training group. Comparisons with other research should consider the starting point and position for the sprint, as anthropometrics can condition sprint performance (36). In our study, the front foot was positioned 20 cm behind the first photocell, and limited torso leaning was allowed. In the running cycle, the hamstrings contract eccentrically during the late swing phase to slow the forward motion of the lower leg and foot (6, 7). This deceleration phase shortens, and forward momentum increases, as running velocity increases, thereby augmenting the demand for eccentric hamstring activation (8). Within this context, eccentric training adaptations could optimize hamstring contractility during the deceleration phase, thereby improving readiness to generate an optimal hip extension moment at ground contact. In addition, hamstring strength plays a fundamental role in sprint performance, as supported by post-intervention speed improvements (15, 37). The significant change in linear sprint observed in the current study following moderate and low volumes of Nordic curl training indicates that the program volume was sufficient to elicit significant effects on speed in elite youth soccer players, although they, as top-tier athletes, may already be near their theoretical limit for adaptation (15). Therefore, it seems that the 8-week low-volume Nordic curl training program, along with typical soccer training (e.g., technical-tactical drills) and the competitive schedule followed during this period, may have been sufficient to elicit changes in sprint performance in this study population. Considering that players' level and physical condition have important implications for their data interpretation and management (38, 39), further studies should delve deeper into the subsequent design of conditioning training programs.

The present results also indicated that a lower Nordic curl volume was more effective in improving CoD performance than moderate or high volumes. It is important to note that participants can improve at these tests by getting older, becoming faster, and practicing them repeatedly. However, this effect is common to all participants across the three groups, thereby minimizing potential between-group biases in the analysis. In this regard, all groups showed a significant improvement in CoD outcomes from baseline to post-intervention. Similarly, prior studies have shown that sub-elite and elite young male soccer players can improve their CoD performance by 2.7%–5.5% with Nordic curl training at low volumes (23), whether in isolation (40) or in combined training programs (41). Rapid CoD ability is critical in soccer, as the game frequently demands sharp turns and tempo shifts that place substantial metabolic and mechanical stress on players (41). In decelerations, eccentric force production supports hip extensor torque, promotes dynamic stabilization of the knee and trunk, and aids the accumulation and reuse of elastic energy in the last ground contact of CoD actions (42). In contrast, straight-line running relies primarily on concentric propulsive action of the lower-limb agonist muscles (43). Given these reasons, strength and conditioning professionals may consider low-volume Nordic curl training as a tool to improve hip and knee deceleration and stabilization during CoD in youth soccer players.

The different outcomes observed across the tests could be explained by the specific physical and mechanical demands of each task. CoD involves repeated deceleration, dynamic stabilization, and reacceleration, all of which require substantial neuromuscular involvement, including hamstrings eccentric force production (8). Although the hamstrings also contribute eccentrically during linear sprinting, especially in the late swing phase, technical and mechanical factors also play a role; therefore, CoD tasks place greater emphasis on braking capacity and neuromuscular control than linear sprinting (6–8). Furthermore, within the sprint, the 10 m sprint performance is more dependent on initial acceleration capacity, to which hamstring force production contributes more directly, whereas performance over longer linear sprint distances may depend relatively more on other determinants beyond neuromuscular factors, such as sprint mechanics, step characteristics, and lower-limb stiffness (44). Because Nordic curl training primarily affects neuromuscular factors and sprint mechanics are less specifically affected, differences in volume prescriptions may not have differentially influenced the 20 m and 10 m sprints, while the different volumes have differentially influenced the CoD. In this regard, it seems that higher Nordic volumes do not confer additional functional gains, while increasing soreness and residual fatigue, potentially interfering with the quality of concurrent soccer-specific and speed training (16). This may explain the greater improvements observed in CoD performance following the low-volume Nordic curl program.

## Limitations and further research

This study has important strengths, including its sample size and the devices used; however, some limitations are noted. First, a control group was included given the nature of the study and its participants, which may limit understanding of the underlying mechanisms. However, the club staff was interested in obtaining the hypothesized performance benefits for all the players. Therefore, future studies should compare these three volumes with a control group without Nordic curl. Second, the relatively short training program duration, volume, and exact exercise technique must be considered when comparing with previous research. Furthermore, the current outcomes should be applied only to the studied context and not beyond it, especially given that retention of improvements after the intervention was not assessed. It would be important that future studies explore different weekly volume distributions (e.g., 1 vs. 2 vs. 3 sessions/week) while keeping total volume constant. The order of the tests was designed according to previous research (27, 28) and was consistent from pre- to post-intervention and among groups, therefore, minimizing potential biases. However, we cannot exclude a possible transfer between tests. Finally, we did not measure strength adaptations to the resistance training program or monitor internal football training load and match participation (minutes played, position, etc.), which may influence improvements in sprint and CoD. Therefore, future studies should include measures of strength (e.g., eccentric strength) and muscle architecture to relate structural changes to performance.

## Conclusion

In-season Nordic curl training enhances linear sprint and CoD in young healthy soccer athletes. In terms of the differences between the three volumes evaluated (low, moderate, and high): (1) low-volume Nordic curl training improved CoD performance, with and without the ball, to a greater extent than high- and moderate-volume programs; (2) both moderate- and low-volume Nordic curl training further improved acceleration (10 m sprint) compared to the high-volume Nordic curl training, with no differences between low and moderate volumes; and (3) all three volumes improved the 20 m sprint, without significant differences between them.

As CoD and sprint performance are key determinants of competitive performance in soccer, this study's findings should be incorporated into routine training practice. From an applied perspective, low-volume Nordic curl training could be more time-efficient, potentially produce less fatigue, and offer clearer benefits than moderate- and high-volume protocols for improving CoD performance, with similar benefits for improving sprint performance. Therefore, a low-volume Nordic curl training program seems to be a more valuable tool than moderate- or high-volume Nordic curl training for youth soccer players.

## Data Availability

The raw data supporting the conclusions of this article will be made available by the authors, without undue reservation.
